# The relationship between asymptomatic organ damage, and serum soluble tumor necrosis factor-like weak inducer of apoptosis (sTWEAK) and Interleukin-17A (IL-17A) levels in non-diabetic hypertensive patients

**DOI:** 10.1186/1471-2369-15-159

**Published:** 2014-10-01

**Authors:** Ihsan Ates, Nihal Özkayar, Fatma Akyel, Canan Topcuoglu, Serdar Akyel, A Nurdan Barça, Fatih Dede

**Affiliations:** Department of Nephrology, Ankara Numune Education and Research Hospital, Sıhhiye, Ankara 061100 Turkey; Biochemistry Department, Ankara Numune Education and Research Hospital, Sıhhiye, Ankara 061100 Turkey; Cardiology Department, Ankara Numune Education and Research Hospital, Sıhhiye, Ankara 061100 Turkey; Radiology Department, Ankara Numune Education and Research Hospital, Sıhhiye, Ankara 061100 Turkey

**Keywords:** Asymptomatic organ damage, Hypertension, Interleukin-17, Tumor necrosis factor-like weak inducer of apoptosis

## Abstract

**Background:**

This study aimed to measure the serum soluble tumor necrosis factor-like weak inducer of apoptosis (sTWEAK) and interleukin-17A (IL-17A) levels in hypertensive patients with/without asymptomatic organ damage (AOD), as well as to determine the relationship between the serum sTWEAK and IL17-A levels, and carotid intima media thickness (CIMT), proteinuria, retinopathy, and the left ventricle mass index (LVMI).

**Methods:**

The study included 159 patients diagnosed with and followed-up for primary hypertension (HT); 79 of the patients had AOD (61 female and 18 male) and 80 did not (52 female and 28 male). sTWEAK and IL-17A levels were measured in all patients.

**Results:**

The sTWEAK level was significantly lower in the patients with AOD than in those without AOD (858.4 pg/mL vs. 1151.58 pg/mL, P = 0.001). The sTWEAK level was negatively correlated with the mean microalbuminuria level and LVMI. The median IL-17A level was significantly higher in the patients with AOD than in those without AOD (2.34 pg/mL vs. 1.80 pg/mL, P = 0.001). There was a positive correlation between mean IL-17A level, and mean microalbuminuria level, CIMT, and LVMI. Multivariate logistic regression analysis showed that patient age, sTWEAK level, and mean 24-h systolic blood pressure were predictors of AOD.

**Conclusions:**

The sTWEAK level was lower and IL-17A level was higher in the patients with AOD. It remains unknown if sTWEAK and IL-17A play a role in the pathophysiology of AOD. Prospective observational studies are needed to determine the precise role of sTWEAK and IL-17A in the development of target organ damage.

## Background

Hypertension (HT) is a major risk factor for cardiovascular events (e.g. stroke, heart attack, sudden death, heart failure, and peripheral artery disease) and end-stage renal disease [1]. In addition to accelerated atherosclerosis, HT causes organ damage, including left ventricle hypertrophy, renal dysfunction, arterial aneurysm, and retinopathy, resulting in morbidity and mortality. Although HT is a serious disease, its etiopathogenesis remains largely unknown. While historically an emphasis has been placed on the role of salt intake, obesity, insulin resistance, the renin-angiotensin system, and the sympathetic nervous system in the etiopathogenesis of HT [2], endothelial dysfunction and immune mechanisms have recently become the focus of greater attention [3]. In addition, recent studies have examined the association between HT, and T lymphocytes and cytokines [4–7].

Tumor necrosis factor-like weak inducer of apoptosis (TWEAK) is a member of the tumor necrosis factor (TNF) superfamily that was first described in 1997 [8]. As other members of the TNF superfamily, TWEAK has multiple forms: mTWEAK, which is bonded to the membrane, and sTWEAK, which is a soluble variant produced following proteolytic cleavage by endoprotease furin. Both of these forms are biologically active [9]. sTWEAK binds to fibroblast growth factor-inducible molecule 14 (Fn14) receptors, and plays a role in cellular proliferation, migration, apoptosis, differentiation, angiogenesis, and inflammation via the nuclear factor κB (NF-κB) pathway [10].

In 2005 interleukin-17 (IL-17) was known as a cytokine produced only by T helper 17 [11], but more recent studies have shown that it can be produced by macrophages, dendritic cells, natural killer cells, natural killer T cells, and γδ T cells. There are 6 known types of IL-17 (IL-17A-F); the prototype is IL-17A [5]. IL-17A is known to play a role in autoimmune and inflammatory diseases, including rheumatoid arthritis, psoriasis, multiple sclerosis, asthma, and inflammatory bowel disease [12, 13], and in chronic vascular inflammation, atherosclerosis, and hypertensive vascular changes [14]. The literature does include studies on sTWEAK and IL-17A in patients with HT [4, 5, 7], but does not include any studies on the relationship between sTWEAK and IL-17A, and asymptomatic organ damage (AOD) in HT patient.

As such, the present study aimed to measure serum levels of sTWEAK and of IL-17A in patients with primary HT -with/without AOD- as well as to determine the relationship between the serum sTWEAK and IL17-A levels, and such markers of AOD as carotid intima media thickness (CIMT), proteinuria, retinopathy, and the left ventricular mass index (LVMI).

## Methods

This study was conducted at the Ankara Numune Education and Research Hospital, Nephrology Clinic, Ankara, Turkey, between July 2013 and November 2013. The study was performed in accordance with the Declaration of Helsinki and the study protocol was approved by the Ankara Numune Education and Research Hospital Ethics Committee. All the patients provided written informed consent to participate in the study.

The study included 159 patients aged >18 years that were previously diagnosed as HT; 79 patients with AOD (18 male and 61 female) (based on grade III-IV retinopathy, LVMI >95 g/m^2^ for females and >115 g/m^2^ for males, CIMT >0.9 mm or plaque, microalbuminuria (30–300 mg/24-h) and 80 patients without AOD (28 male and 52 female). Patients with secondary HT, dyslipidemia (low-density lipoprotein [LDL] cholesterol >130 mg/dL, triglycerides [TG] >150 g/dL), diabetes mellitus, acute renal injury, nephrotic proteinuria, coronary heart disease, heart failure, peripheral artery disease, cerebrovascular events, malignancy, liver disease, rheumatic diseases, thyroid diseases, and cigarette and alcohol use were excluded.

Duration of HT (years) was calculated based on patient self-reports of the date they were first diagnosed as HT to the date of inclusion in the study. The types of antihypertensive drugs the patients used included renin angiotensin system (RAS) blockers, calcium channel blockers (CCB), beta blockers, and diuretics.

The WatchBP 03 device (Microlife WatchBP AG, Switzerland) was used for 24-h ambulatory blood pressure monitoring (ABPM). Patients were informed about the procedure and the device; they were instructed to perform their daily activities as normal, but to remain immobile while the device was recording measurements. The devices were programmed to record blood pressure measurements every 15 min from 07.00 to 23.00 (daytime) and every 20 min from 23.00 to 07.00 (nighttime). The method was considered reliable if >70% of measurements were valid.

### Biochemical parameters

Blood samples for analysis of biochemical parameters were collected in the morning following a 12-h fasting. Collected blood was immediately centrifuged at 4000 rpm for 10 min to separate the plasma and serum, and serum was stored at -80°C until analyzed. All biochemical parameters were analyzed in the same serum sample. Patients were asked to collect 24-h urine samples; they were instructed not to collect urine from the first urination after waking the morning of the day they started to collect the urine, to urinate into a collection container during every urination thereafter, including the first urination upon waking the following morning, and to bring all the collected urine to the laboratory.

Photometric analysis of hemoglobin was performed using a Sysmex XE 2100 (Roche Diagnostics Corp. Indiana, USA) hematology auto-analyzer, 24-h urine protein was measured via the microalbumin turbidimetric method, creatinine and total protein were measured using the albumin colorimetric method, uric acid, fasting glucose, TG and total cholesterol were measured via the enzymatic colorimetric method, and high-density lipoprotein (HDL) cholesterol was measured via the homogenous enzymatic colorimetric method using a Hitachi Modular P800 (Roche Diagnostics Corp. Indiana, USA) auto-analyzer. LDL cholesterol was calculated using the Friedewald method [15]. The GFR (glomerular filtration rate) was calculated according to the simplified version of the Modification of Diet in Renal Disease Study prediction equation formula, as follows: GFR = 186 × creatinine^– 1.154^ × age^– 0.203^ × 1.212 (if African ‒ American) × 0.742 (if female) [16].

### sTWEAK measurement

Serum sTWEAK (pg/mL) was measured using a serum ELISA kit (eBioscience, Human TWEAK Instant ELISA, cat. no. BMS2006INST). The calculated overall intra-assay and inter-assay coefficients of variation were 7.9-9.2%.

### IL-17A measurement

Serum IL-17A (pg/mL) was measured using a serum ELISA kit (eBioscience, human IL-17A platinum ELISA, cat. no. BMS2017). The calculated overall intra-assay and inter-assay coefficients of variation were 7.1-9.1%.

### Fundoscopic examination

Into both eyes of each patient, 1 drop of tropicamide 1% solution was administrated 15 min prior to funduscopic examination. Funduscopic examination was performed using a biomicroscopy device (Topcon SL-3C, Topcon Corporation, Japan). Retinopathy staging was performed in accordance with ESC/ESH 2013 hypertension guidelines, as follows: grade I: focal or general arteriolar narrowing; grade II: arteriovenous nicking; grade III: retinal hemorrhages, microaneurysms, hard exudates, and cotton wool spots; grade IV: grade III signs and papilledema and/or macular edema) [1].

### Echocardiographic examination

Echocardiography was performed by an investigator blinded to each patient’s clinical status, using a commercial echocardiography machine (Vivid 7, GE Vinged Ultrasound AS, Horten, Norway) equipped with a 2.5-MHz phased array transducer. Complete 2D, color, pulsed, and continuous-wave Doppler examinations were performed according to standard techniques. The LVMI was calculated from 2D echocardiographic measurements, using the Devereux formula, and was indexed to body surface area, as follows: LVMI = 1.04 × [(IVST + PWT + LVDd)3 – (LVDd)3] – 13.6 [17].

### Carotid ultrasonography

CIMT was measured with patients in the supine position with their hand bent backwards. One radiologist that was blinded to the clinical status of the patients used a high-resolution B-mode ultrasound device (Logic 7, GE Med. Inc., USA), a linear probe on the right and left common carotid artery, and an automated system. Measurements were made at 3 points: the right and left common carotid artery, bifurcation, and first 2 cm of the internal carotid artery. Longitudinal measurements were made from the distances defined as vessel lumen echogenicity and media-adventitia echogenicity. Mean CIMT was calculated by averaging 3 measurements made at each carotid artery [18].

### Statistical analysis

Statistical analysis were performed using Statistical Package for Social Sciences (SPSS) v.20.0 for Windows (SPSS, Inc., Chicago, IL). Kolmogorov-Smirnov test was used to analyze data with normal distribution. Numeric parameters with normal distribution are presented as mean ± standard deviation (SD) and numeric parameters without normal distribution are presented as median. Categorical variables are expressed as number and percentage. Correlations between numeric parameters were analyzed via Pearson’s and Spearman’s correlation analysis. The chi-square test and Fisher’s exact chi-square test were used to compare categorical variables. Between-group differences in numeric parameters with normal distribution were analyzed using the independent samples t test, and between-group differences in numeric parameters without normal distribution were analyzed using the Mann–Whitney U test. The effect of risk factors associated with AOD alone was analyzed via univariate logistic regression analysis. Significant variables based on logistic regression analysis were incorporated into a multivariate logistic regression model. ROC (receiver operating characteristic) curve analysis was used to determine the value of the serum sTWEAK level in predicting AOD. To determine the prediction point the point with the highest sensitivity and specificity, and the lowest false positive and false negative predictive points were selected. The level of statistical significance was set at P < 0.05

## Results

The study included 159 patients; 79 patients with AOD (18 male and 61 female; mean age: 54.7 ± 12.0 years) and 80 patients without AOD (28 male and 52 female; mean age: 49.4 ± 11.5 years). There weren’t any significant differences in demographic data between the 2 groups, except for mean age and median duration of HT (P < 0.05). Patients in both groups used RAS blockers, CCBs, beta blockers, and diuretics as antihypertensive drugs. Although mean 24-h systolic blood pressure (SBP) and 24-h diastolic blood pressure (DBP) were significantly higher in the AOD group, there weren’t any significant differences between groups according to the type of antihypertensive drugs used (P > 0.05). Demographic and laboratory findings in both groups are presented in Table 1.

**Table 1:** **Patient demographic and laboratory findings**

Variables	All patients	Patients with AOD	Patients without AOD	P
n (%)	159	79 (49.6%)	80 (50.4%)	
Age (years)	52.02 ± 12.1	54.71 ± 12.0	49.36 ± 11.5	0.005*
Gender				0.115
Female, n (%)	113 (71.1)	61 (77.2)	52 (65)	
Male, n (%)	46 (28.9)	18 (22.8)	28 (35)	
BMI (kg/m^2^)	29.55 ± 4.7	30.04 ± 4.7	29.05 ± 4.6	0.195
Duration of HT (years)	3.74 (4)	5 (3)	2.5 (4)	0.037*
Antihypertensive drugs				
Class (n,%)				
RAS blocker	86 (54.1)	45 (57.0)	41 (51.2)	0.470
Beta blocker	28 (17.6)	17 (21.5)	11 (13.8)	0.198
CCB	63 (39.6)	35 (44.3)	28 (35.0)	0.230
Diuretic	54 (34)	26 (33.8)	28 (36.8)	0.691
CIMT (mm)	0.75 ± 0.17	0.80 ± 0.2	0.68 ± 0.1	0.001*
LVMI (g/m^2^)	90.05 ± 21.1	100.57 ± 21.1	78.64 ± 13.9	0.001*
Microalbuminuria (mg/d)	10 (15,8)	19.32 (41,7)	7.17 (6,9)	0.001*
Proteinuria (mg/d)	94.98 (70.4)	111.25 (114.8)	84.25 (59.1)	0.001*
Retinopathy (n,%)				0.002*
None	91 (57.2)	39 (49.4)	52 (65.0)	
Grade I	28 (17.6)	13 (16.5)	15 (24.6)	
Grade II	28 (17.6)	15 (19.0)	13 (21.3)	
Grade III	12 (7.5)	12 (15.2)	-	
sTWEAK (pg/mL)	961.12 (560.9)	858.4 (365.2)	1151.58 (598.4)	0.001*
IL-17A (pg/mL)	2.1 (0,9)	2.34 (0,9)	1.80 (0,7)	0.001*
24-h SBP (mmHg)	124.31 ± 14.7	129.53 ± 16.8	119.16 ± 9.9	0.001*
24-h DBP (mmHg)	77.83 ± 9.8	79.53 ± 11.8	76.15 ± 6.9	0.029*

Numeric parameters are shown as mean ± SD and median (interquartile range).

Categorical parameters are shown as n (%).

*Denotes a significant difference between the groups (P < 0.05).

In evaluation of all patients for types of antihypertensive drugs used, means of 24 –h SBP and 24-h DBP for patients received RAS blocker, CCB, beta blocker and diuretics were similar (P > 0.05).

**Figure 1:**
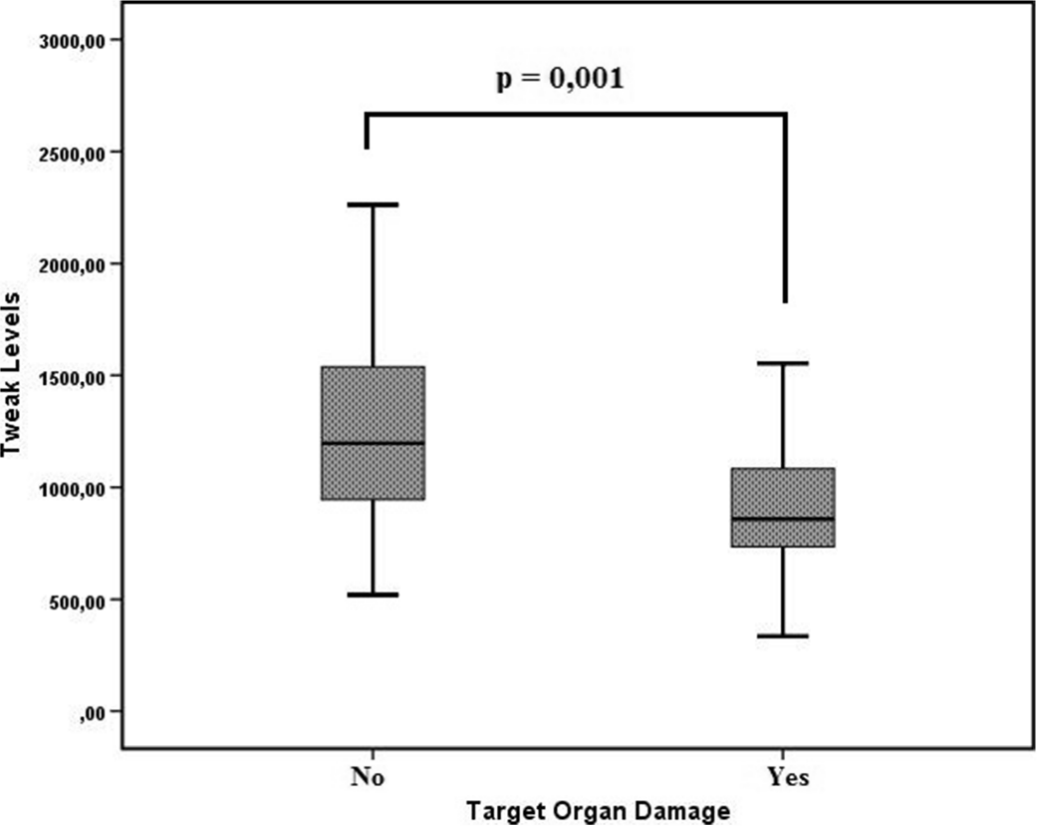
**Between-group comparison of the serum sTWEAK level.**

**Figure 2:**
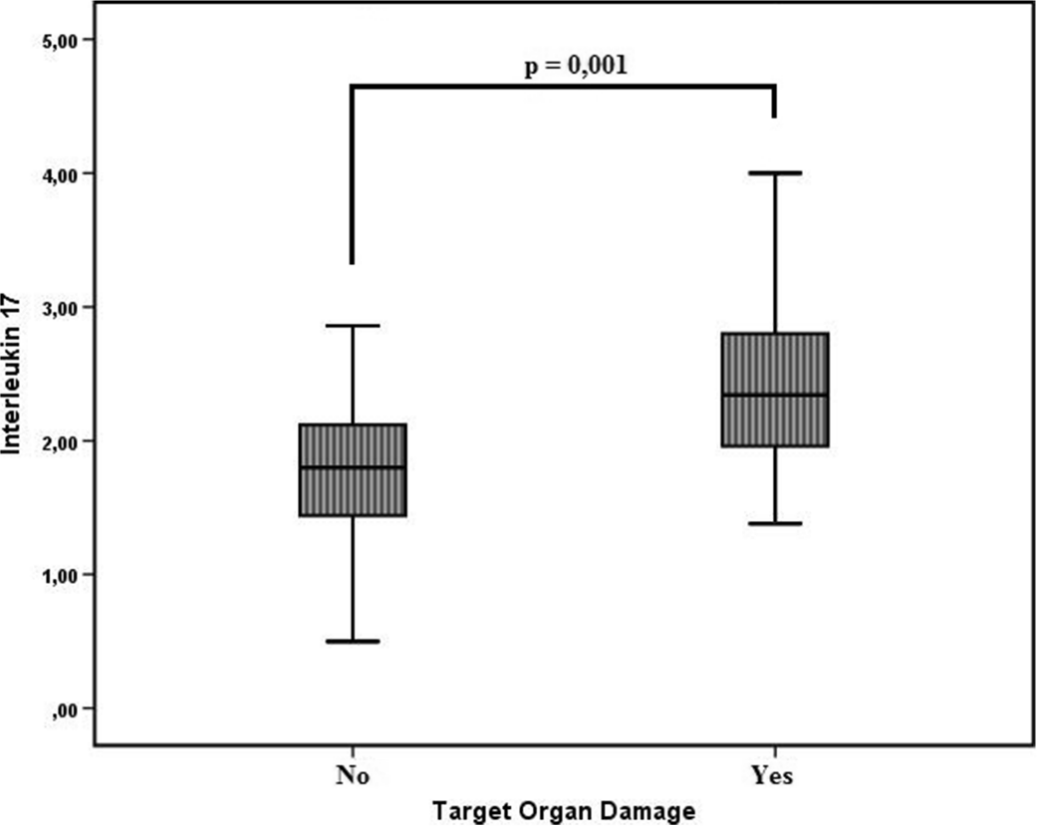
**Between-group comparison of the serum IL-17A level.**

Mean 24-h SBP and 24-h DBP were significantly higher in the AOD group (24-h SBP: 129.5 mmHg ± 16.8 mmHg vs. 119.2 mmHg ± 9.9 mmHg, P = 0.001; 24-h DBP: 79.5 mmHg ± 11.8 mmHg vs. 76.1 mmHg ± 6.9 mmHg, P = 0.029). Median serum sTWEAK was significantly lower in the AOD group (858.4 pg/mL vs. 1151.58 pg/mL, P = 0.001) (Figure 1). Median IL-17A was significantly higher in the AOD group (2.34 pg/mL vs. 1.80 pg/mL, P = 0.001) (Figure 2).

The serum sTWEAK level was negatively correlated with the microalbuminuria level, proteinuria level, LVMI, mean 24-h SBP, and duration of HT (P < 0.05); however, the serum sTWEAK level was not correlated with CIMT, retinopathy stage, or mean 24-h DBP (P > 0.05). There was a weak negative correlation between the serum sTWEAK level and patient age (r = -0.183, P = 0.021).

There was a positive correlation between the IL-17A level, and the microalbuminuria level, proteinuria level, CIMT, LVMI, and mean 24-h SBP (P < 0.05); however, retinopathy stage and mean 24-h DPB were no correlated with the IL-17A level (P > 0.05). There was a weak positive correlation between the IL-17A level and patient age (r = 0.184, P = 0.020, and a moderately negative correlation between the serum sTWEAK level and IL-17A level (r = -0.354, P < 0.05).

Mean age of HT patients with AOD was significantly higher than of those without AOD (54.71 ± 12.0 vs 49.36 ± 11.5; p = 0.005). The age factor was also included in the regression model and thus it is determined that decreased sTWEAK level independent of age increased the risk for AOD (OR = 0.985, p = 0.002).

Mean 24-h SBP was positively correlated with the microalbuminuria level, proteinuria level, CIMT, and LVMI (P < 0.05); however, retinopathy stage and mean 24-h SBP were not correlated (P > 0.05). In addition, there wasn’t a correlation between mean 24-h SBP and patient age (r = -0.027, P = 0.733). There was a positive correlation between mean 24-h DBP, and the microalbuminuria level and CIMT (P < 0.05); however, retinopathy stage was not correlated with proteinuria or LVMI (P > 0.05). Table 2 shows the correlation analyses in detail. Retinopathy stage ≥ III, microalbuminuria, proteinuria, LVMI, and CIMT findings were not included in the logistic regression analysis, as they were used to identify target organ damage.

**Table 2:** **Correlation analysis of sTWEAK, IL-17A, 24-h SBP, and 24-h DBP, and the risk factors**

Variables	sTweak		IL17-A		24-h SBP		24-h DBP	
	r	P	r	P	r	P	r	P
Microalbuminuria	-0.188	0.024*	0.253	0.002*	0.430	0.001*	0.241	0.004*
Proteinuria	-0.165	0.048*	0.193	0.023*	0.195	0.019*	0.144	0.084
CIMT	-0.104	0.340	0.229	0.036*	0.366	0.001*	0.217	0.043*
LVMI	-0.264	0.001*	0.327	0.001*	0.283	0.001*	0.143	0.082
Retinopathy Grade	-0.137	0.130	0.120	0.193	0.079	0.387	-0.021	0.815
24-h SBP	-0.186	0.019*	0.300	0.001*	-	-	0.769	0.001*
24-h DBP	-0.057	0.475	0.146	0.070	0.769	0.001*	-	-
sTWEAK	-	-	-0.354	0.001*	-0.186	0.019*	-0.057	0.475
IL-17A	-0.354	0.001*	-	-	0.300	0.001*	0.146	0.070
Duration of HT	-0.159	0.045*	0.117	0.144	0.050	0.532	-0.121	0.130
Age	-0.183	0.021*	0.184	0.020*	-0.027	0.733	-0.252	0.001*

*Denotes a significant correlation (P < 0.05).

**Figure 3:**
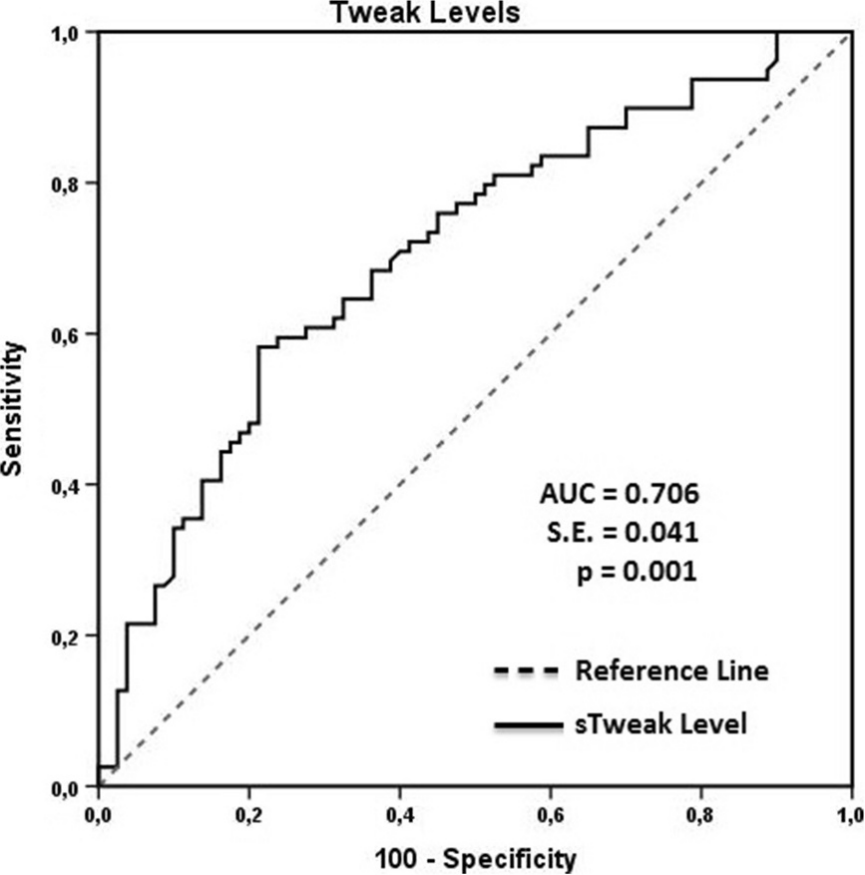
**ROC curve analysis of the prediction of AOD based on the serum sTWEAK level.**

Patient age, duration of HT, and mean sTWEAK, IL-17A, 24-h SBP, and 24-h DBP were associated with AOD and were analyzed via univariate logistic regression, and the significant risk factors were included in the multivariate logistic regression model. Multivariate logistic regression analysis showed that patient age (OR = 1.045, P = 0.012), the sTWEAK level (OR = 0.985, P = 0.002), and mean 24-h SBP (OR = 1.065, P = 0.001) were predictive of AOD (Table 3). A serum sTWEAK level ≤ 877.78 pg/mL predicted AOD with sensitivity of 58.23% and specificity of 78.75% (AUC = 0.706 ± 0.041 (SE); 95% CI: 0.629-0.775; P < 0.001) (Figure 3). Age >59 years predicted AOD with sensitivity of 45.24% and specificity of 76.25% (AUC = 0.590 ± 0.045(SE); 95% CI: 0.519-0.670; P = 0.027). A mean 24-h SBP level >127 mmHg predicted AOD with sensitivity of 49.38% and specificity of 73.75% (AUC = 0.603 ± 0.042(SE); 95% CI: 0.524-0.682; P = 0.003). The AUC value for the sTWEAK level predictive of AOD was higher than that for age and 24-h SBP (24-h SBP vs. sTWEAK: AUC: 0.103, P = 0.033; SBP vs. age: AUC: 0.013, P = 0.806; sTWEAK vs. age: AUC: 0.116, P = 0.028). The present findings indicate that the serum sTWEAK level was a better diagnostic predictor of AOD than patient age and mean 24-h SBP.

**Table 3:** **Logistic regression analysis of the risk factors associated with AOD**

Variables	Univariate		Multivariate	
	OR (95% CI)	P	OR (95% CI)	P
Age	1.040 (1.011-1.070)	0.006*	1.045 (1.010-1.076)	0.012*
Duration of HT	1.062 (1.046-1.143)	0.042*	0.995 (0.911-1.087)	0.236
sTWEAK	0.990 (0.985-0.994)	0.001*	0.985 (0.983-0.996)	0.002*
IL-17A	1.138 (1.030-1.393)	0.021*	0.996 (0.805-1.232)	0.495
24-h SBP	1.061 (1.032-1.091)	0.001*	1.065 (1.030-1.093)	0.001*
24-h DBP	1.038 (1.020-1.075)	0.034*	0.943 (0.875-1.017)	0.125

Nagelkerke R^2^ = 0.343, P < 0.05.

*P < 0.05 is considered significant for statistical analyses.

## Discussion

In the present study the serum sTWEAK level was significantly lower and the IL-17A level was significantly higher in the HT patients with AOD than in those without AOD. To the best of our knowledge the present study is the first to investigate the relationship between AOD in HT patients, and the serum sTWEAK and IL-17A levels.

Recent studies indicate that HT and AOD associated with HT not only occur due to hemodynamic disturbances, but that the natural and adaptive immune systems play a role [19]. Several rat studies reported that following infusion of deoxycorticosterone acetate and angiotensin II, a number of cytokines (TNF-*a*, IL-1B, IL-6, IL-10, IL-17, and TGF-B1) were synthesized by the natural and adaptive immune systems, resulting in vascular inflammation [5, 20]. Although many cytokines are known to play a role in the etiopathogenesis of HT, the relationship between HT, and IL-17A and sTWEAK remains unclear.

TWEAK is a cytokine of the TNF family that activates many components of cellular activity, including cell reproduction and cell death [20]. Some studies that investigated the sTWEAK level reported that it was low in patients with acute coronary syndrome [21], heart failure [22], coronary artery disease [23], chronic renal disease [24], and kidney transplantation [25]. Various explanations for low sTWEAK levels in such patients have been posited, including that in cases of chronic inflammation the Fn14 level increases and binds to sTWEAK, resulting in a decrease in the serum sTWEAK level [26], and that an increase in the scavenger receptor CD 163 level causes inflammatory macrophages to break down sTWEAK in the presence of inflammation [27].

A search of the literature indicated that Karadurmus et al.’s [7] study is the only one to have examined the relationship between the serum sTWEAK level and HT. Their study included 51 patients that were recently diagnosed as HT and treatment naïve; the mean sTWEAK level in the HT patients was lower than that in the healthy control group, but the relationship between the sTWEAK level and AOD was not investigated. In the present study the serum sTWEAK level was observed to be lower in the HT patients AOD than in those without AOD. Sustained high blood pressure can lead to chronic inflammation, resulting in target organ damage, such as atherosclerosis and proteinuria. The lower sTWEAK level in the present study’s HT patients with AOD might have been due to chronic inflammation that already existed.

An earlier study reported that high levels of Fn14 in patients with cardiac hypertrophy was associated with elevated blood pressure and that the sTWEAK level was lower in the cardiac hypertrophy patients because of binding to Fn14 receptor [28]. Another study reported elevated Fn14 levels in patients with non-immune primary glomerular disease and proteinuria [29]. In the present study there was a negative correlation between the serum sTWEAK level, and microalbuminuria level, proteinuria level, LVMI, and mean 24-h SBP, which might have been due to high level of Fn14 that bound to sTWEAK and decrease in the serum sTWEAK level following chronic inflammation.

IL-17A is a cytokine that is suggested to play a role in the pathophysiology of HT, and its relationship to HT has been the subject of recent research. Several recent studies have reported that the IL-17A level increases in cases of atherosclerosis and vascular inflammation, and that such increases contribute to progression of inflammation [14, 30].

Nguyen et al. reported that SBP increased significantly in mice that received IL-17A (IP injection of 1 μg/d for 7 d), which was attributed to a decrease in the response to aortic nitric oxide (NO)-dependent relaxation and an increase in NO synthase Thr495 phosphorylation (which leads to a decrease in NO production and vasodilation, and is associated with HT) [5].

Madhur et al. [31] conducted a prospective study that included 112 HT patients, and reported that the IL-17 level was significantly higher in the HT patients than in the normotensive group. They suggested that the finding was due to an increase in the level of Th17 in T cells and an increase in the IL-17 level following infusion of chronic angiotensin II, an increase in superoxide radicals due to deposition of IL-17 on vessel walls, a decrease in endothelium-related vasodilatation, and some affected genes (e.g. stanniocalcin-1), which caused HT and vascular inflammation. In addition, they also reported that IL-17 cell infiltration in the aorta occured in cases of angiotensin II-induced HT and that HT was not maintained in IL-17 knockout mice.

Liu et al. [32] observed a positive correlation between the IL-17A level and CIMT in atherosclerotic patients. In the present study the serum IL-17A level was significantly higher in HT patients with AOD than in those without AOD. In addition, there was a positive correlation between the serum IL-17A level and the microalbuminuria level, proteinuria level, CIMT, LVMI, and mean 24-h SBP, all of which are indicators of AOD. A decrease in NO related to IL-17A may lead to endothelial dysfunction that can result in atherosclerosis and an increase in CIMT, as reported by Madhur and Nguyen.

A study that investigated the sTWEAK level as a predictor of target organ damage in HT patients reported that a low sTWEAK level was an independent risk factor for cardiovascular events [33]; however, findings concerning the predictive value of the IL-17A level are inconsistent.

The literature includes studies that have investigated the relationship between a variety of biomarkers and AOD in patients with HT [34–36]. Morillas et al. [34] reported that risk factors in HT patients, including age, smoking, diabetes mellitus, waist circumference, IL-6, and sTNF-R1 (marker of inflammation and apoptosis), are independent predictors of target organ damage based on multivariate logistic regression analysis. Ratto et al. [35] reported that the hs-CRP level was an independent predictor of AOD, based on multivariate logistic regression analysis, and Çalışkan et al. [36] observed that the serum uric acid level was a good predictor of coronary flow reserve based on ROC curve analysis (AUC = 0.760, P < 0.0001). In the present study the sTWEAK level was observed to be a better predictor of AOD than the other predictors (e.g. age and SBP) (AUC = 0.706, P < 0.001).

The present findings show that patient age, sTWEAK, and mean 24-h SBP were independent predictors of AOD, based on a multivariate model of risk factors (age, sTWEAK level, IL-17A level, mean 24-h SBP and 24-h DBP, and duration of HT) that independently play a role in target organ damage. Mean age of the present study’s HT patients with AOD was significantly higher than of those without AOD, and there was a weak negative correlation between the serum sTWEAK level and patient age; however, regression analysis with inclusion of age showed that the relationship between the serum sTWEAK level and AOD was independent of age. To the best of our knowledge the present study is the first to report on the relationship between the serum sTWEAK level and age. The only limitation of the present study is its cross-sectional design, which precludes any conclusion regarding the causal relationship between the serum sTWEAK and IL17-A levels, and AOD in HT patients.

## Conclusion

As AOD is an interim stage in the progression of vascular diseases and is a pathology with an important role in identifying cardiovascular risk in HT patients, AOD in HT patients must be carefully examined. Additional prospective observational studies are needed to more clearly delineate the role of sTWEAK and IL-17A in the development of target organ damage.

## Abbreviations

AOD: Asymptomatic organ damage
CCB: Calcium channel blocker
RAS: Renin angiotensin system
CMIT: Carotid intimal media thickness
Fn14: Fibroblast growth factor-inducible molecule 14
GFR: Glomerular filtration rate
HDL: High-density lipoprotein
HT: Hypertension
IL-17A: Interleukin-17A
LVMI: Left ventricular mass index
LDL: Low-density lipoprotein
NO: Nitric oxide
sTWEAK: Soluble tumor necrosis factor-like weak inducer of apoptosis
TG: Triglycerides
24-h SBP: 24-h systolic blood pressure
24-h DPB: 24-h diastolic blood pressure.
